# Thermoelectricity in vertical graphene-C_60_-graphene architectures

**DOI:** 10.1038/s41598-017-10938-2

**Published:** 2017-09-15

**Authors:** Qingqing Wu, Hatef Sadeghi, Víctor M. García-Suárez, Jaime Ferrer, Colin J. Lambert

**Affiliations:** 1 Quantum Technology Centre, Lancaster University, LA1 4YB Lancaster, United Kingdom; 2 Departamento de Física, Universidad de Oviedo, 33007 Oviedo, Spain; 3 Nanomaterials and Nanotechnology Research Center (CSIC-Universidad de Oviedo), El Entrego, 33940 Asturias Spain

## Abstract

Recent studies of single-molecule thermoelectricity have identified families of high-performance molecules. However, in order to translate this discovery into practical thin-film energy-harvesting devices, there is a need for an understanding of the fundamental issues arising when such junctions are placed in parallel. This is relevant because controlled scalability might be used to boost electrical and thermoelectric performance over the current single-junction paradigm. As a first step in this direction, we investigate here the properties of two C_60_ molecules placed in parallel and sandwiched between top and bottom graphene electrodes. In contrast with classical conductors, we find that increasing the number of parallel junctions from one to two can cause the electrical conductance to increase by more than a factor of 2. Furthermore, we show that the Seebeck coefficient is sensitive to the number of parallel molecules sandwiched between the electrodes, whereas classically it should be unchanged. This non-classical behaviour of the electrical conductance and Seebeck coefficient are due to inter-junction quantum interference, mediated by the electrodes, which leads to an enhanced response in these vertical molecular devices.

## Introduction

**Figure 1:**
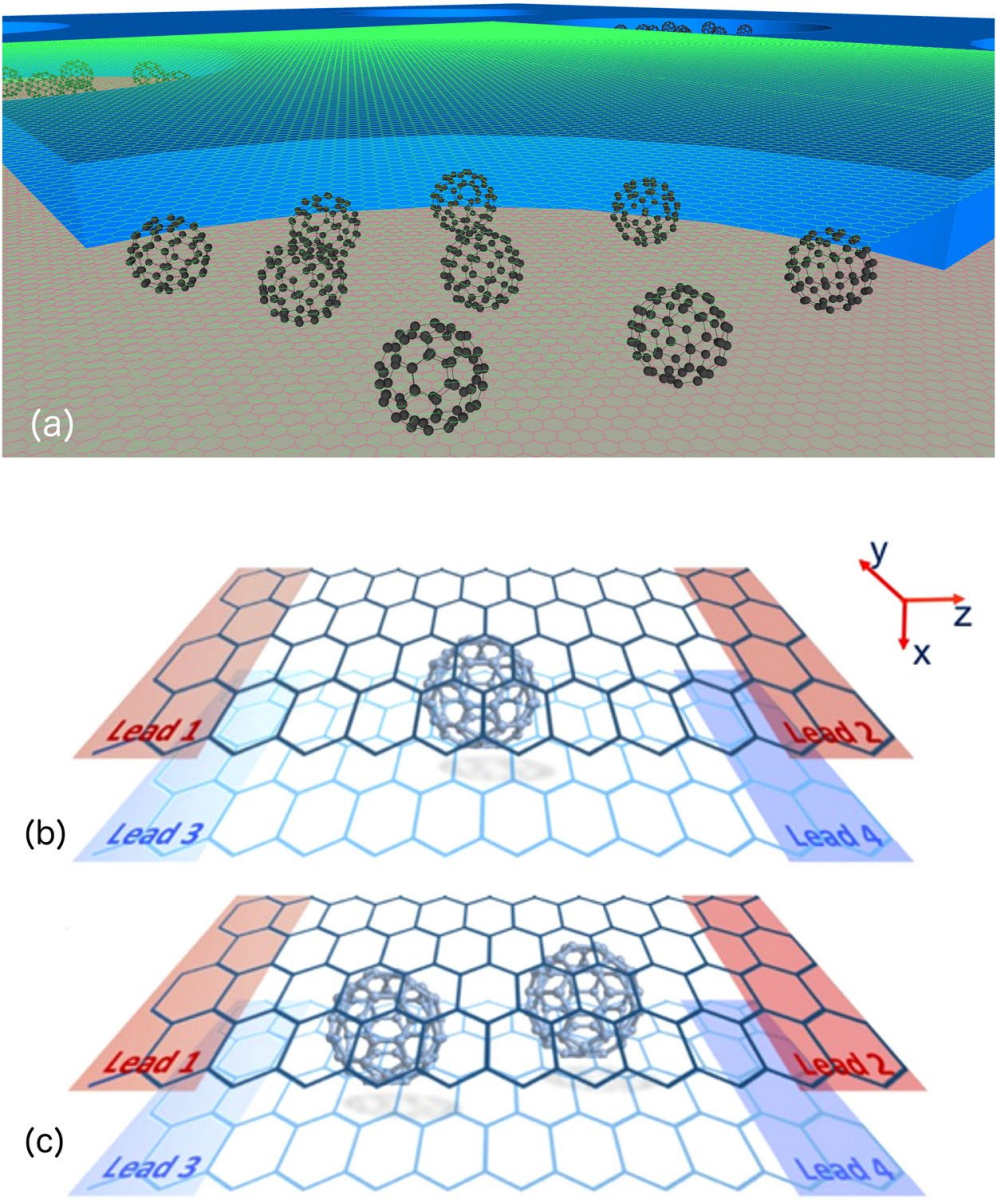
(**a**) Vertical scalable concept for molecular thermoelectricity. An insulating spacer is placed on top of a graphene bottom electrode, and drilled with nanopores. These pores are filled with C_60_ molecules. A top graphene electrode is deposited. Thermoelectricity is enhanced by Quantum Interference. Schematics of the two simulated devices: (**b**) a C_60_-monomer and (**c**) a C_60_-dimer sandwiched between two graphene monolayers.

Molecular devices consisting of single or multiple molecules bridging two or more electrodes have attracted intense theoretical and experimental interest, due to their tuneable and unique transport properties, including negative differential resistance (NDR)^1–3^ electrical switching^4–6^ and thermoelectric power generation^7–13^. The conversion of a temperature gradient Δ*T* to a voltage difference Δ*V* is controlled by the Seebeck coefficient *S* = −Δ*V*/Δ*T*. Common inorganic thermoelectric materials such as Pb, Bi, Co, Sb are toxic and expensive due to limited global sources. Therefore, in recent years, different strategies have been proposed to exploit the thermoelectric properties of nanostructured organic materials or organic molecules^14–18^. At the single-molecule level, the Seebeck coefficient S can be controlled using a gate electrode in a three-terminal device^19^. Furthermore, the sign and magnitude of *S* can be changed by modulating the coupling of the molecule to electrodes using the pressure induced by a STM tip^9^. Additionally, it has been demonstrated that the Seebeck coefficient of molecular junctions can be enhanced by manipulating the intermolecular interactions of C_60_ molecules placed in series between two gold electrodes^10^. This effect arises from the quantum mechanical origin of thermopower at the molecular scale. Indeed at a qualitative level, if the transmission probability of electrons with energy *E* passing from one electrode to another through a C_60_ molecule is *T*_1_(*E*), the transmission probability through two C_60_ molecules placed in series is approximately proportional to *T*_1_(*E*)^2^. Consequently, (after averaging over a quantum phase) the conductance of two molecules placed in series *G*_2_ is equal to ${G}_{1}^{2}$, whereas Ohm’s law predicts *G*_2_ = *G*_1_/2. Similarly, the Mott formula $S\propto -\frac{\partial \,\mathrm{ln}\,T(E)}{\partial E}{|}_{E={E}_{F}}$, predicts that the thermopower coefficients are related by ${S}_{2}\propto 2\times {S}_{1}$, whereas classically *S*_2_ should be the same with *S*_1_. The aim of the present paper is to determine if similar non-classical behaviour occurs when molecules are placed in parallel between two electrodes as in the concept device shown in Fig. 1(a). If two such molecules in parallel behave classically, then the electrical conductance doubles according to Ohm’s law and the Seebeck coefficient is unchanged. Consequently, if many molecules were placed in parallel to form a self-assembled monolayer (SAM), then the electrical conductance would be proportional to the number of molecules and the Seebeck coefficient would be insensitive to the number of parallel molecules bridging the junction. Reuter and co-workers have addressed how the electrical conductance of two molecules placed in parallel between two electrodes need not be 2*G*_1_, and have proposed that deviations from Ohm’s law are a signature of direct inter-molecular interactions or ‘cooperativity’^20^. In what follows, our aim is to examine this expectation from a microscopic point of view by computing the change in thermoelectric properties when two C_60_ molecules are placed in parallel between two graphene electrodes. Our results demonstrate that even when there is no direct inter-molecular coupling, indirect inter-molecular interactions mediated by the graphene electrodes produce quantum interference effects in the electronic structure of the molecular junction. As a consequence, the Seebeck coefficient is sensitive to the number *N* of parallel molecules and the electrical conductance is not simply proportional to *N*. These indirect interactions, if controlled properly can boost the electrical and thermoelectric performance of a device over the single-molecule paradigm.

## Results

**Figure 2:**
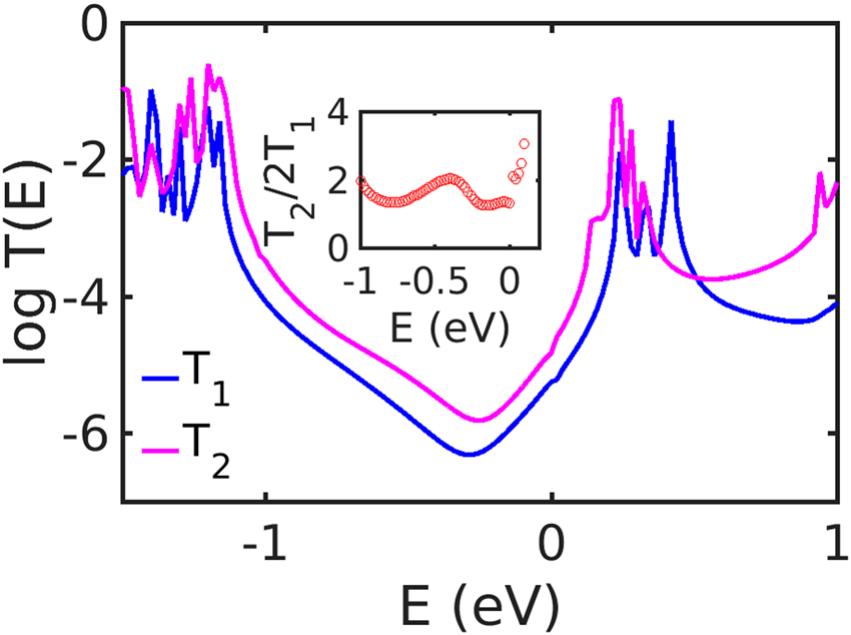
Transmission coefficients of electrons of energy *E* passing from electrode 1 to electrode 4. The blue and pink curves show the transmission coefficients *T*_1_(*E*) and *T*_2_(*E*) of the C_60_-monomer and -dimer devices, respectively. The inset figure shows the ratio of the two transmission coefficients.

We have designed the vertical four-terminal devices shown in Fig. 1(b) and (c), where a single C_60_ and a C_60_ dimer are sandwiched between two horizontal graphene sheets, respectively. The two graphene sheets are separated by an optimized vertical distance and are electronically decoupled, except via the transport path though the buckyball(s) from the top to the lower sheet. The transmission functions *T*_1,2_(*E*) for electrons of energy *E* passing from the top-left electrode (lead 1) to the bottom-right electrode (lead 4) are shown in Fig. 2 for the C_60_-monomer and -dimer devices^21^. As expected, due to the periodic boundary conditions chosen for the electrodes, the number of open channels for both devices is 2 in the energy range between −1.5 and +1.5 eV around the Fermi energy *E*_*F*_. The energy position of the HOMO- and LUMO-mediated (highest-occupied and lowest unoccupied molecular orbital, respectively) resonances does not depend on the relative position and orientation of the molecule and the graphene electrodes for van der Waals chemical bonding^22^. We therefore predict that *T*_1,2_(*E*) in Fig. 2 should remain qualitatively the same as the C_60_ molecules move around and rotate. Furthermore, a close match between graphene’s Fermi energy and the C_60_ HOMO or LUMO resonances is predicted since both are carbon-based materials^23,24^,. We find that the LUMO resonances are much closer to the Fermi energy than the HOMO-mediated ones for the vertical junctions shown in Fig. 1, a result consistent with previous studies^25–27^. Furthermore, the conductance gap (e.g.: the gap between the HOMO and LUMO resonances) for the dimer device shrinks due to the splitting of the degenerate HOMOs and LUMOs, a quantum interference effect caused by their indirect coupling mediated by the electrodes. In order to estimate the departure from Ohm’s law caused by this indirect inter-molecular interaction, we show in the inset plot in Fig. 2 the ratio *T*_2_/(2*T*_1_) over an energy range within the conductance gap. This ratio is approximately 1.5 in the energy range between −0.8 eV and 0 eV and then increases quickly above the Fermi energy when approaching the LUMO resonance. This increase above unity breaks Ohm’s law and is a consequence of the quantum interference effect that modifies the conductance gap. Indirect inter-molecular coupling not only changes the conductance ratios, but also affects the slope of the logarithm of the transmission coefficients. At low-enough temperatures, the Seebeck coefficient can be obtained using Mott formula^7^

1 $$S=-\frac{{\pi }^{2}}{3}\frac{{k}_{B}^{2}T}{|e|}\,{\partial }_{E}\,\mathrm{ln}\,T(E{)|}_{E={E}_{F}}$$

Consequently, the Seebeck coefficient is proportional to the slope of natural logarithm of transmission function at the Fermi level and since these slopes differ, the Seebeck coefficients of the monomer and dimer junctions are different.

**Figure 3:**
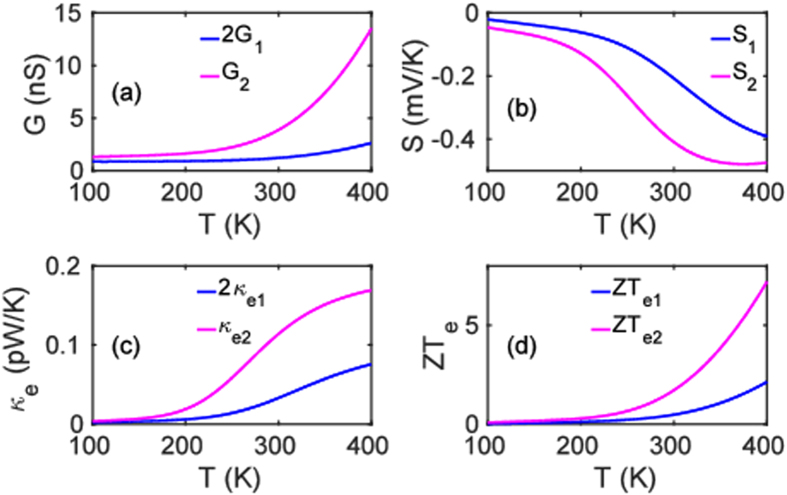
Temperature evolution of the thermoelectric coefficients: (**a**) Electrical conductance *G*(*T*); (**b**) Seebeck coefficients $S(T)$ and (**c**), (**d**) electronic contribution to thermal conductance ${\kappa }_{e}(T)$ and figure of merit $Z{T}_{e}(T)$. The pink and blue curves correspond to the dimer and twice the monomer devices, respectively.

**Figure 4:**
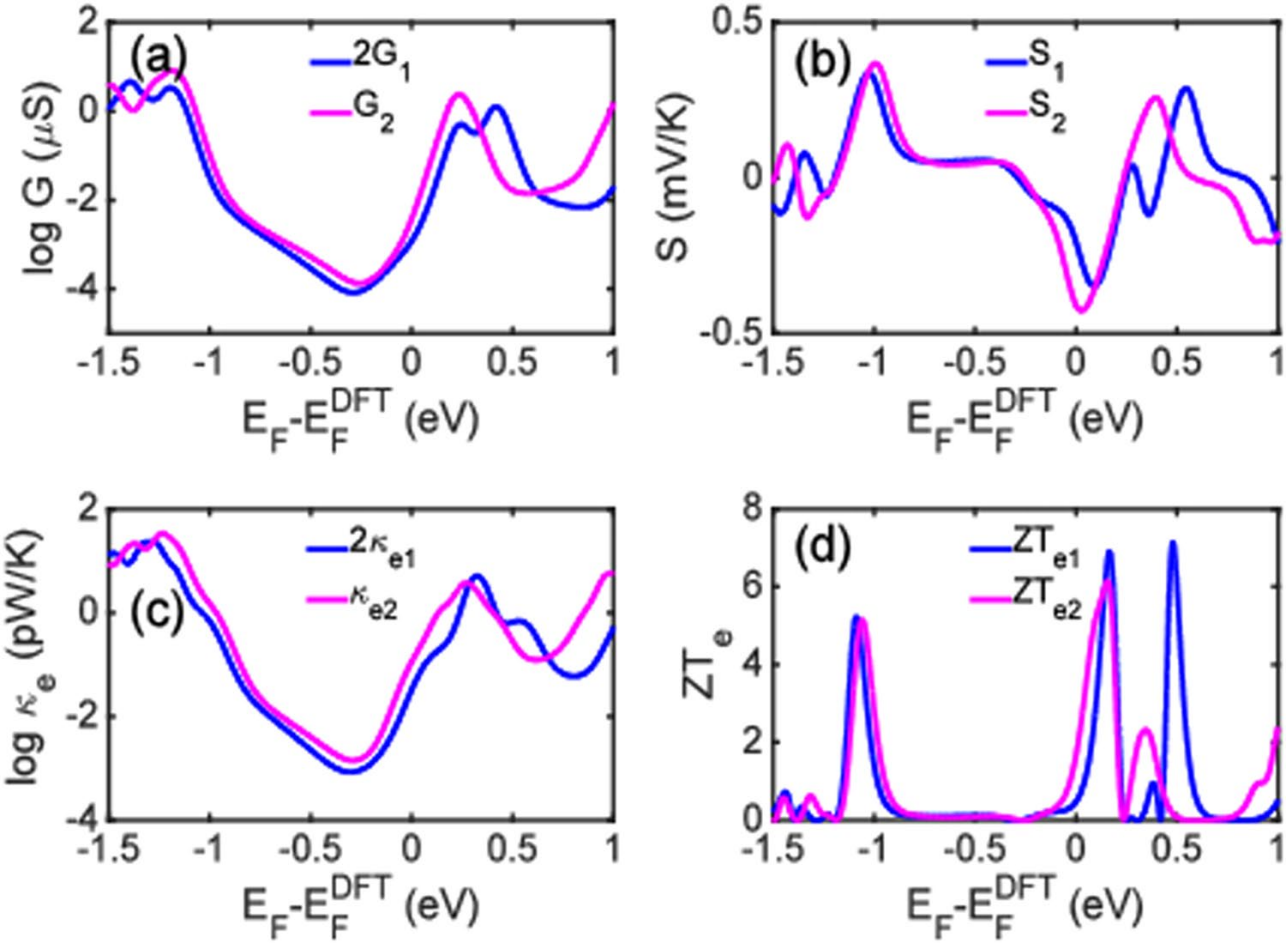
(**a**) Electrical conductance $G$; (**b**) Seebeck coefficients $S$ and (**c**), (**d**) electronic contribution to thermal conductance ${\kappa }_{e}$ and figure of merit $Z{T}_{e}$ as a function of the Fermi level at room temperature. The blue and pink curves represent the dimer and twice the monomer devices.

Figure 3 demonstrates that *G*, *S*, the electronic contribution to the thermal conductance *κ*_*e*_ and the electronic contribution to the thermoelectric figure of merit $Z{T}_{e}={S}^{2}GT/{\kappa }_{e}$ behave non-classically over a wide range of temperatures. Figure 3(a) shows that the electrical conductance of the dimer system (pink curve) is more than twice the conductance of the monomer (blue curve) over a wide temperature range. Figure 3(b) reveals that the Seebeck coefficient of the dimer junction is higher than that of the monomer junction. Figure 3(d) shows that although the electronic contribution to the thermal conductance (Fig. 3(c)) of the dimer junction is higher than that of the monomer device, the higher electrical conductance and Seebeck coefficient of the dimer junction combine to deliver a higher electronic figure of merit. Since the Fermi energy of the electrodes may be changed by doping or external gating, we analyze in Fig. 4 the quantum behaviour of the thermoelectric properties of the junctions as a function of the Fermi energy at room temperature. Figure 4(a) and (c) demonstrate that both the electrical conductance and the electronic contribution to the thermal conductance of the dimer device are more than twice those of the monomer junction over an energy window around the Fermi energy. Figure 4(b) reveals that the dimer configuration also has a larger absolute value of the Seebeck coefficient than the monomer in the energy range between −0.2 and 0.1 eV and also from 0.3 to 0.5 eV. Figure 4(d) demonstrates that *ZT*_*e*_ is also larger in the vicinity of *E*_*F*_.

**Figure 5:**
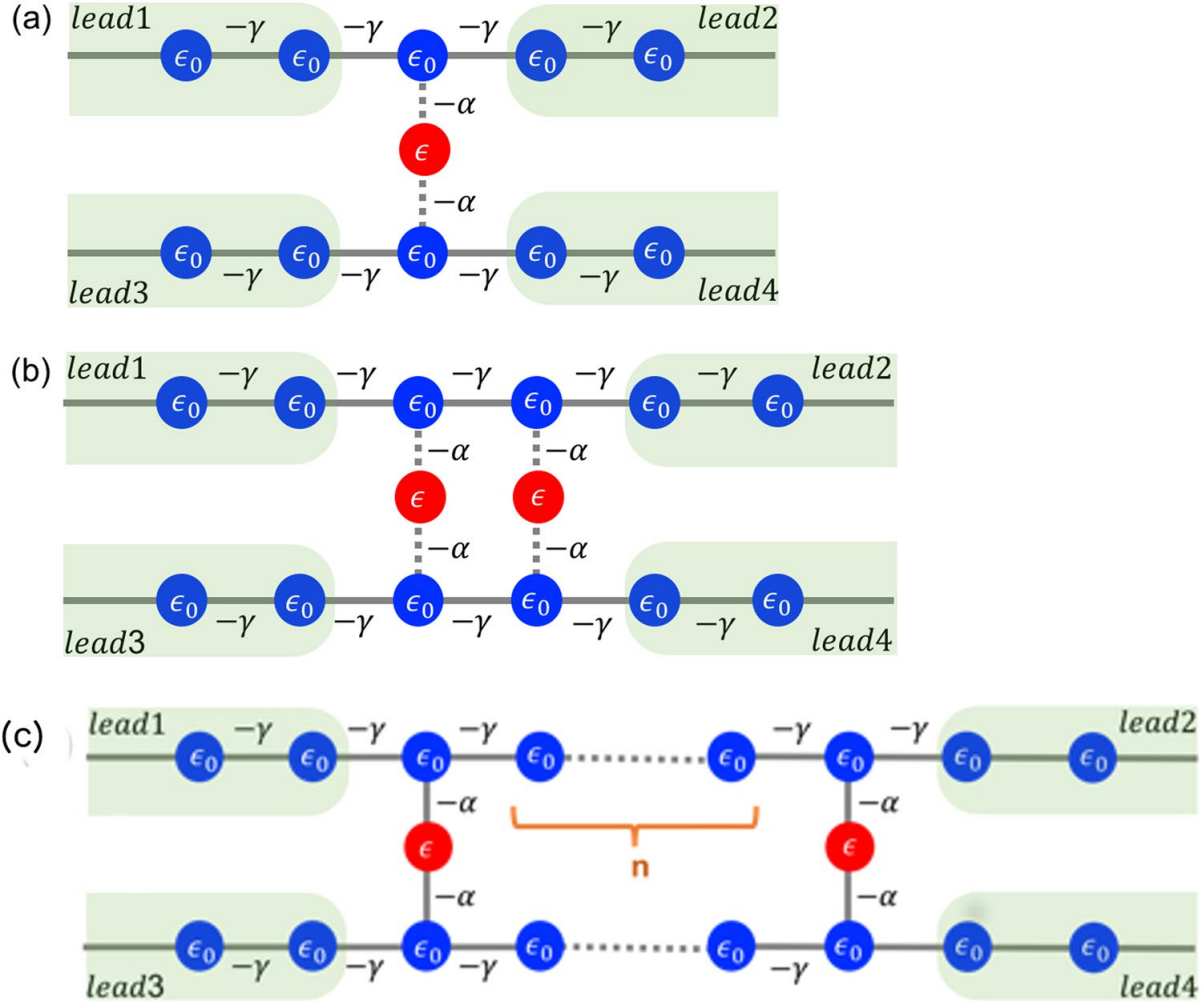
Tight binding models having (**a**) a One-atom pathway; (**b**) Two-atom pathways. The chains represent the graphene electrodes, where the blue dots correspond to carbon atoms; the red dots represent the C_60_ molecules. To fix the energy scale, the hopping integral *γ* between blue sites is set to unity. The hopping integral *α* between a red and a blue site is set to 0.2. The hopping integral between red sites in (**b**) is set to zero. The on-site energy of both blue and red sites *ε*_0_ and *ε* is set to 0.25. This reflects the relevant energy level landscape found in the DFT calculation and displayed in Fig. 2. (**c**) A modification of two-site model in (**b**), where *n* represents the separation between red sites evaluated by the number of blue sites lying in between.

In order to obtain further insight into this quantum interference effect, we have performed calculations of model tight-binding structures that share similar parallel electron pathways to the devices shown in Fig. 1. The schematics of those model structures is shown in Fig. 5. The red balls representing C_60_ molecules are sandwiched between the blue chains representing the graphene electrodes. There is no direct hopping between the two red sites in the two-site model as in the C_60_-dimer DFT calculation. Therefore, any departure from Ohm’s law must be due to the indirect coupling between the two sites arising from quantum interference via the electrodes.

**Figure 6:**
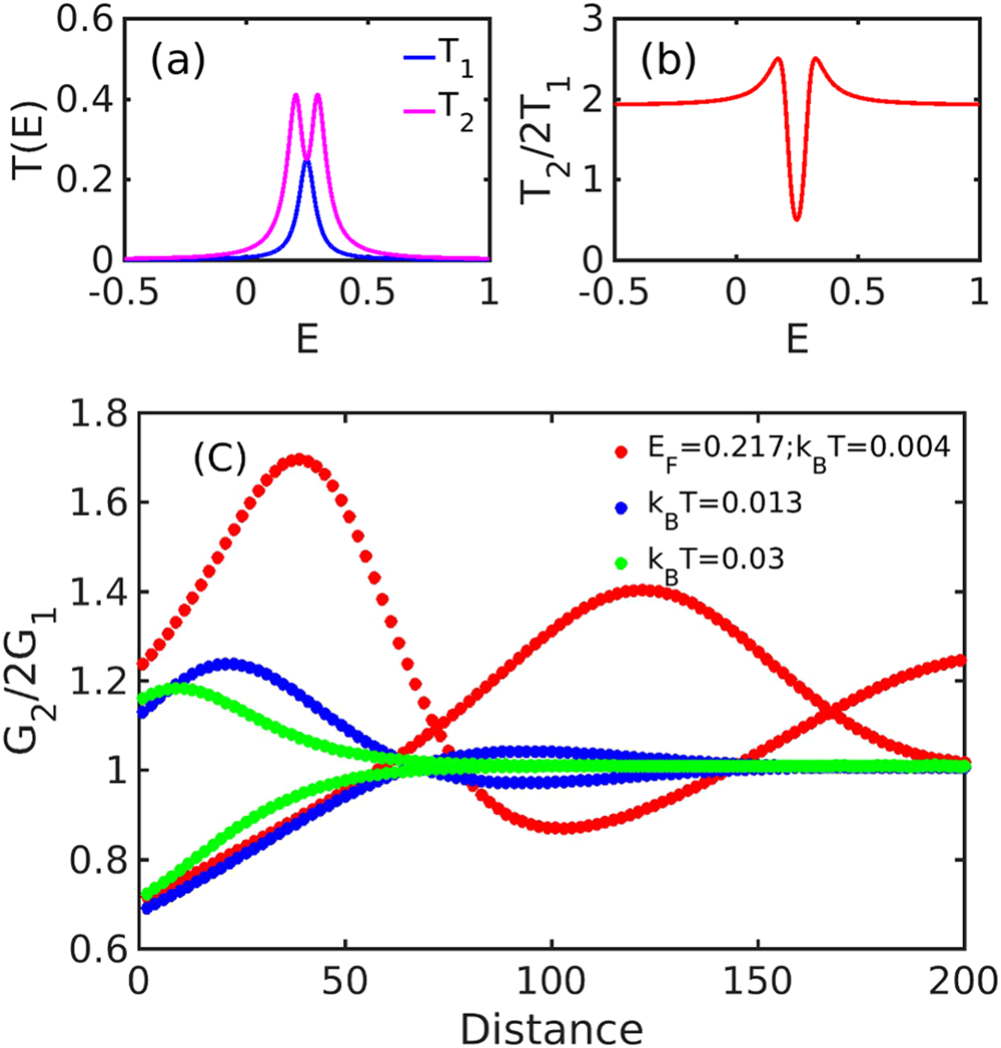
(**a**) Transmission coefficients as a function of energy *E* for the one-site and two-site models shown in Fig. 5; The blue line stands for ${T}_{1}$; (**b**) Ratio *T*_2_(2*T*_1_). (**c**) Conductance ratio ${G}_{2}\mathrm{/(2}\,{G}_{1})$ evaluated at ${E}_{F}=0.217$ and different temperatures: ${k}_{B}\,T=0.004$, 0.013 and 0.03 (red, blue and green lines, respectively); The two curves at each temperature show the conductance ratio for even and odd n.

The transmission coefficients *T*_2_, *T*_1_ and the ratio *T*_2_/(2*T*_1_) of these models are displayed in Fig. 6(a). The pink curve shows that for the two-site model, the transmission resonance at energy *E* = *ε* is split in two due to the indirect coupling via the electrodes. The ratio *T*_2_/(2*T*_1_), shown in Fig. 6(b) varies from approximately 0.5 to 2.5 and converges to a constant value around 2, which is twice the expected classical value of unity. Consequently, despite the simplicity of the tight binding model, we find that it captures the mechanism underlying the quantum interference effects uncovered in the DFT-based analysis.

We now discuss how this quantum interference behaviour depends on the distance between the two sites. To do so, we modify the model shown in Fig. 5(b) and displace the two red sites laterally so that there are *n* blue sites in between them as shown in Fig. 5(c). Figure 6(b) shows the conductance ratio ${G}_{2}\mathrm{/2}\,{G}_{1}$ at three different temperatures (low, intermediate and high). We find that the ratio varies periodically with the distance *n*, and that it has an envelope that decreases with increasing *n*. We also find an even-odd quantum interference effect in the conductance ratio as a function of the parity of *n*. We therefore plot two curves for each temperature, one for even *n* and another for odd *n*. The Figure also shows that this low-temperature oscillatory behaviour is damped as the temperature increases. This damping is controlled by the temperature-dependent phase coherence length, and can be understood straightforwardly from the standard expression of the conductance $G={G}_{0}\,{L}_{0}$, where *G*_0_ is the conductance quantum unit and the integral *L*_0_ is defined in Eq. (1). The integration window for *L*_*n*_ covers an energy range of order ${k}_{B}\,T$ that is centered at the Fermi energy. Because the period of the oscillations in *T*(*E*) are energy-dependent and the different contributions to the integral dephase, the conductance oscillations die away at values of *n* beyond a certain dephasing length that is inversely proportional to the temperature, as illustrated in Fig. 6. Interestingly, in the absence of inelestic scattering, the asymptotic ratio ${G}_{2}\mathrm{/2}\,{G}_{1}$ does not approach unity for large distances *n*, even though the oscillations disappear above a certain temperature. This is because the asymptotic ratio depends on the position of the Fermi energy relative to the quantum interference-split resonance shown in Fig. 6(a). The above behaviour is analogous to that of AlGaAs/GaAs quantum rings, where the temperature-dependent phase coherence length, extracted from Aharonov-Bohm magnetoresistance measurements, decreases as the temperature rises above 2.0 K^28^. The result also agrees well with that obtained by magneto-transport experiments combined with weak localization theory in MgZnO thin film^29^, where the phase coherence length varies from 38.4 nm to 99.8 nm when temperature declines from 50 K to 1.4 K.

## Conclusion

The electrical conductance *G*_2_ of two parallel C_60_ molecules sandwiched between two graphene monolayers does not follow Ohm’s law, because it is more than twice larger than the conductance *G*_1_ of a single C_60_ molecule. This non-classical behaviour is due to indirect inter-molecular quantum interference effects mediated by the electrodes. Furthermore, increasing the number of C_60_ molecules sandwiched in parallel between graphene monolayers from one to two also increases the Seebeck coefficient, which is another non-classical effect. This is significant because it demonstrates that single-molecule thermoelectric properties will not translate into thin-film materials formed from self-assembled monolayers in a classical manner and by exploiting quantum interference, the thermoelectric performance of such SAMs can exceed classical expectations. Further insight into this quantum interference effect is gained by analyzing a tight binding model that features parallel electron transport through two sites. The model predicts that the thermoelectric properties of the dimer will oscillate with the dimer separation *n* up to a phase coherence length, which decreases with increasing temperature.

## Methods

The vertical four-terminal devices shown in Fig. 1(b) and (c) are assigned periodic boundary conditions in the x and y directions. Furthermore, to eliminate edge effects in the z direction, the four regions (labelled leads 1–4) are semi-infinite crystalline leads, which channel electrons to and from reservoirs placed at infinity. In the case of the C_60_-dimer, the horizontal distance between the nearest atoms of the two C_60_s is initially set to 6 Å to avoid direct coupling between the buckyballs. We have used the DFT code SIESTA^30^ to obtain the optimized geometry adopting the local density approximation and the Ceperley-Alder functional for exchange and correlation. We have also chosen a double-z plus polarization (DZP) basis set. After relaxing this structure using about a thousand molecular dynamics steps of 1 fs, this distance is changed by only a fraction of an Å. In this situation, examination of the pseudo-atomic-orbital-based hamiltonian describing the molecules confirms that there is no direct interaction between the two C_60_ molecules. We have extracted the resulting mean-field Hamiltonian and overlap matrices and used them to compute the electrical and thermoelectric properties of the devices with our transport code GOLLUM^31^. The transmission coefficient for electrons of energy *E* travelling from lead *i* to lead *j* is calculated through the standard expression^32^

2 $${T}_{ij}(E)={\rm{Tr}}\,[{{\rm{\Gamma }}}_{{\rm{i}}}\,{{\rm{G}}{\rm{\Gamma }}}_{{\rm{j}}}\,{{\rm{G}}}^{\dagger }]$$

where G(E) is the device’s retarded Green’s function and ${{\rm{\Gamma }}}_{i}(E)$ is the imaginary part of the self-energy ${{\rm{\Sigma }}}_{i}$ of electrode *i*. The thermoelectric coefficients including the electrical conductance *G*, the thermopower *S*, the electronic contribution to the thermal conductance ${\kappa }_{e}$ and to the figure of merit $Z{T}_{e}$ can be written using standard text-book formulae^32,33^.

3 $$G(T)={G}_{0}\,{L}^{0}$$

4 $$S(T)=-\frac{1}{e\,T}\,\frac{{L}^{1}}{{L}^{0}}$$

5 $${\kappa }_{e}(T)=-\frac{2}{h\,T}\,\frac{{L}^{0}\,{L}^{2}\,-\,{({L}^{1})}^{2}}{{L}^{0}}$$

6 $$Z{T}_{e}(T)=\frac{{({L}^{1})}^{2}}{{L}^{0}\,{L}^{2}\,-\,{({L}^{1})}^{2}}$$

in terms of the Lorenz numbers

7 $${L}_{ij}^{n}={\int }_{-\infty }^{\infty }\,dE\,{(E-\mu )}^{n}\,{T}_{ij}(E)\,(-{\partial }_{E}\,f(E))$$

where ${G}_{0}=2{e}^{2}/h$ is the conductance quantum, *h* is Planck’s constant, *e* is the absolute value of electron’s charge, $T=({T}_{i}+{T}_{j}\mathrm{)/2}$ is the mean temperature of electrodes *i* and *j*, *f* is the Fermi-Dirac distribution function and *μ* is the chemical potential of the device at equilibrium, e.g.: when all leads voltages are set to zero. The specific implementation is explained in detail in the reference article of the GOLLUM code^31^.
